# Automated Computer-Assisted Diagnosis of Pleural Effusion in Chest X-Rays via Deep Learning

**DOI:** 10.3390/diagnostics15182322

**Published:** 2025-09-13

**Authors:** Ya-Yun Huang, Yu-Ching Lin, Sung-Hsin Tsai, Tsun-Kuang Chi, Tsung-Yi Chen, Shih-Wei Chung, Kuo-Chen Li, Wei-Chen Tu, Patricia Angela R. Abu, Chih-Cheng Chen

**Affiliations:** 1 Program on Semiconductor Manufacturing Technology Academy of Innovative Semiconductor and Sustainable Manufacturing, National Cheng Kung University, Tainan City 701401, Taiwan; m28124023@gs.ncku.edu.tw (Y.-Y.H.); wctu@gs.ncku.edu.tw (W.-C.T.); 2 Department of Medicine, College of Medicine, Chang Gung University, Taoyuan City 333423, Taiwan; lin0927@cgmh.org.tw; 3 Division of Thoracic Oncology, Department of Respiratory and Critical Care Medicine, Chang Gung Memorial Hospital, Chiayi City 613016, Taiwan; 4 Department of Respiratory Care, Chang Gung University of Science and Technology, Chiayi County 61363, Taiwan; 5 Department of Medical Education, Chang Gung Memorial Hospital Linkou, Taoyuan City 333423, Taiwan; mpq689@cgmh.org.tw; 6 Department of Electrical Engineering, Ming Chi University of Technology, New Taipei City 243303, Taiwan; 7 Department of Electronic Engineering, Feng Chia University, Taichung City 40724, Taiwan; m1208782@o365.fcu.edu.tw; 8 Department of Information Management, Chung Yuan Christian University, Taoyuan City 320317, Taiwan; kuochen@cycu.edu.tw; 9 Department of Electronic Engineering, National Cheng Kung University, Tainan City 701401, Taiwan; 10 Ateneo Laboratory for Intelligent Visual Environments, Department of Information Systems and Computer Science, Ateneo de Manila University, Quezon City 1108, Philippines; pabu@ateneo.edu; 11 Department of Automatic Control Engineering, Feng Chia University, Taichung City 40724, Taiwan; chenccheng@fcu.edu.tw; 12 Department of Aeronautical Engineering, Chaoyang University of Technology, Taichung City 41349, Taiwan

**Keywords:** medical image, image enhancement, chest X-rays, pleural effusion, deep learning

## Abstract

**Background/Objectives:** Pleural effusion is a common pulmonary condition that, if left untreated, may lead to respiratory distress and severe complications. Chest X-ray (CXR) imaging is routinely used by physicians to identify signs of pleural effusion. However, manually examining large volumes of CXR images on a daily basis can require substantial time and effort. To address this issue, this study proposes an automated pleural effusion detection system for CXR images. **Methods:** The proposed system integrates image cropping, image enhancement, and the EfficientNet-B0 deep learning model to assist in detecting pleural effusion, a task that is often challenging due to subtle symptom presentation. Image cropping was applied to extract the region from the heart to the costophrenic angle as the target area. Subsequently, image enhancement techniques were employed to emphasize pleural effusion features, thereby improving the model’s learning efficiency. Finally, EfficientNet-B0 was used to train and classify pleural effusion cases based on processed images. **Results:** In the experimental results, the proposed image enhancement approach improved the model’s recognition accuracy by approximately 4.33% compared with the non-enhanced method, confirming that enhancement effectively supports subsequent model learning. Ultimately, the proposed system achieved an accuracy of 93.27%, representing a substantial improvement of 21.30% over the 77.00% reported in previous studies, highlighting its significant advancement in pleural effusion detection. **Conclusions:** This system can serve as an assistive diagnostic tool for physicians, providing standardized detection results, reducing the workload associated with manual interpretation, and improving the overall efficiency of pulmonary care.

## 1. Introduction

According to the chronic obstructive pulmonary disease (COPD) statistics released by the World Health Organization (WHO) in 2023 [1], approximately 390 million people worldwide are affected by COPD, and this number continues to rise each year. The disease ranks as the third leading cause of death globally and is particularly prevalent in low- and middle-income countries [2]. This situation underscores the fact that many patients face challenges in accessing adequate medical services for the prevention and treatment of lung diseases. Promoting lung health is therefore crucial for improving overall public health, as maintaining healthy lung function not only enhances patients’ quality of life but also reduces the burden on healthcare systems [3]. In this context, raising awareness of the importance of early diagnosis and treatment of lung diseases is of particular significance.

**Figure 1:**
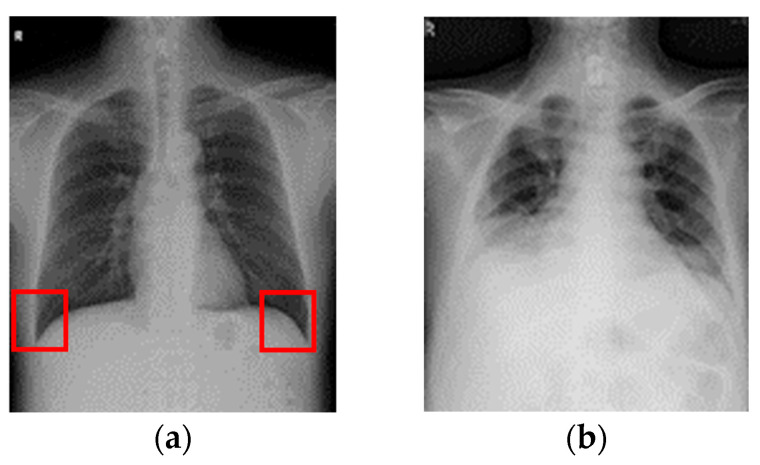
Pleural effusion symptoms on CXR image. (**a**) Absence of pleural effusion symptoms. (**b**) Presence of pleural effusion symptoms.

Medical imaging technologies have played a crucial role in achieving this goal, with chest X-rays (CXR) serving as one of the most widely used diagnostic tools in pulmonary medicine [4]. Among the conditions detectable through CXR, pleural effusion occurs when an abnormal amount of fluid accumulates in the pleural cavity. Under normal physiological conditions, the pleural cavity contains only a small amount of fluid that facilitates smooth movement between the lungs and the chest wall [5]. Excessive fluid accumulation can result in pleural effusion, which is often associated with severe complications and is positively correlated with various lung diseases. It frequently occurs alongside pathological conditions such as pulmonary infections or tumor-induced compression, contributing to critical complications including inflammation [6,7]. CXR imaging enables clinicians to identify fluid accumulation in the lungs, as shown in Figure 1, making accurate detection of pleural effusion a vital step in diagnosis. In Figure 1a, the red box highlights the costophrenic angle, which represents a key region for identifying pleural effusion.

Building upon the importance of CXR imaging in diagnosing pleural effusion, recent advancements in automated medical imaging technologies have demonstrated strong potential to improve both diagnostic efficiency and accuracy in clinical practice [8]. The accelerated progress of artificial intelligence (AI) has introduced new opportunities for enhancing diagnostic precision, accelerating medical image analysis, and improving treatment efficiency [9,10]. Given the high number of CXRs that radiologists must interpret daily, recent research has explored the integration of deep learning techniques for detecting thoracic diseases, including pleural effusion [11]. Convolutional neural networks (CNNs) have achieved notable results in this area, with applications ranging from image segmentation and enhancement for lung disease detection to object detection models that have achieved COVID-19 detection accuracies exceeding 89% [12,13]. Some techniques have already been proposed for the automated detection of pleural effusion in CXR images, including ResNet-18 [5], support vector machines [14], and DenseNet121 [15], which reported accuracies of 77.00%, 87.70%, and 87.18%, respectively. In addition, S. Serte and A. Serener [16] introduced a CNN-based approach for pleural effusion detection in CXR images that achieved an accuracy as high as 91.00%. Beyond single CNN models, ensemble approaches combining multiple CNNs have also been explored to further improve recognition accuracy [17], incorporating architectures such as AlexNet, MobileNet-V2, VGG, ResNet-18, and ResNet-50. However, despite the growing adoption of deep learning in medical imaging, relatively few studies have specifically targeted the automated detection of pleural effusion in CXR images. This gap underscores the need for focused research to develop accurate, reliable, and automated diagnostic tools.

To address this need, the present study proposes a CXR-based analysis method designed to provide radiologists with standardized diagnostic information, thereby automating clinical workflows and reducing inter-observer variability in image interpretation [18,19]. The proposed system integrates an image cropping algorithm, image enhancement techniques, and the EfficientNet-B0 architecture [20] to achieve automated detection of pleural effusion. A summary of the major contributions is provided below:

1. This study employs image segmentation to pre-process lung X-rays, retaining only the lower half of the lungs, from below the heart to the costophrenic angle, thereby enabling the model to focus more effectively on the target region.
2. An effective image enhancement approach is introduced, which improves model performance by 4.33% compared with results obtained without enhancement.
3. The proposed system integrates image cropping, image enhancement, and CNN classification, achieving an accuracy of 927%, representing a substantial improvement of 21.30% over existing studies.

## 2. Methods

**Figure 2:**
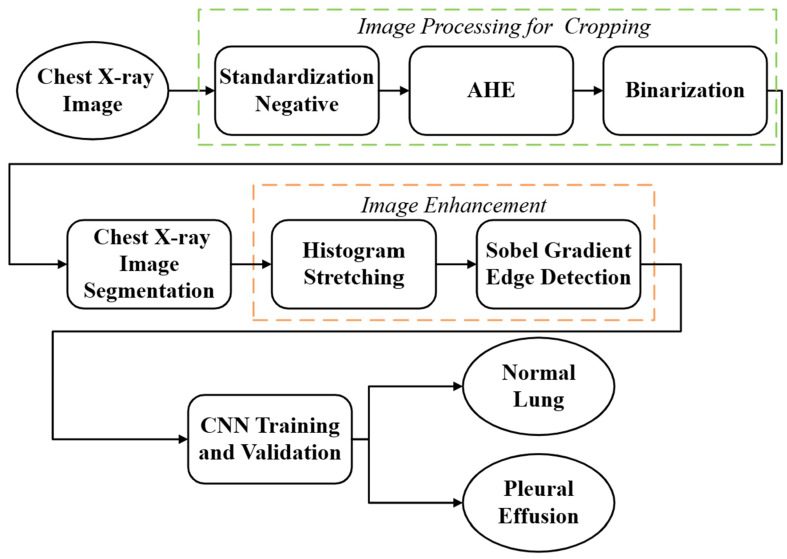
The flowchart of the proposed system for automated detection of pleural effusion in CXR images.

This study proposed a system for detecting pleural effusion in CXR images by integrating various image processing methods with artificial intelligence. To achieve accurate detection, the workflow was divided into four main stages, as shown in Figure 2. First, raw CXR images were input into the system. Image preprocessing was then performed to remove peripheral noise and irrelevant background information. Next, image cropping was applied to retain only the target regions, thereby reducing the influence of excessive non-essential information on model training efficiency. Image enhancement methods were subsequently used to highlight features associated with pleural effusion. Finally, a classical CNN was employed for model training and evaluation. This study focused on developing a computer-aided diagnostic system to support pulmonologists in clinical decision-making by providing more objective and standardized recognition results, ultimately improving patient care efficiency.

### 2.1. Image Preprocessing for CXR Image

However, the costophrenic angle is a key anatomical landmark for examining pleural effusion. Under normal conditions, the structure of the costophrenic angle is clearly visible on CXR images. When pleural effusion occurs, fluid accumulates in these angles, causing them to appear blurred or obscured on CXR images. Additionally, since pleural effusion is influenced by gravity and tends to accumulate in the lower parts of the lungs, this study focused on the lower half of the lung, specifically the region from the heart to the costophrenic angle, as the target area. To further improve the accuracy of region extraction, an image preprocessing method was introduced to enable precise segmentation of the costophrenic angle.

#### 2.1.1. Standardization Negative

**Figure 3:**
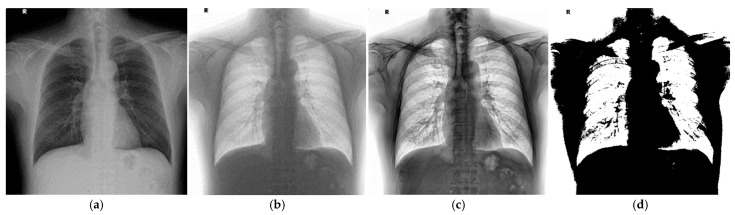
The CXR image preprocessing result. (**a**) Gray scale. (**b**) Negative. (**c**) AHE. (**d**) Binarization.

To enhance the clarity of lung boundaries, the original CXR images were first converted to grayscale before applying further preprocessing, thereby reducing computational complexity. Each pixel was converted based on the weighted average of its red, green, and blue (RGB) channel values. The resulting grayscale image is shown in Figure 3a.

Subsequently, a negative transformation was applied to the grayscale image, as defined in Equation (1). By inverting the grayscale values, darker regions became brighter, while brighter regions became darker, increasing the contrast between the lungs and surrounding tissues. This process effectively enhanced the lung contours, making the lung regions more distinguishable, as shown in Figure 3b.

(1) $$I_{negative}=1-I_{gray}$$

#### 2.1.2. AHE

After grayscale conversion and negative transformation, the lung regions became more visually prominent. To further enhance image contrast and improve the visibility of fine details, adaptive histogram equalization (AHE) was applied to the preprocessed images, as shown in Figure 3c. AHE enhances local contrast by redistributing pixel intensities within small regions of the image, making subtle structures such as lung contours more clearly visible. Compared to conventional histogram equalization, AHE is more effective in handling images with uneven brightness, making it particularly suitable for medical imaging applications.

#### 2.1.3. Binarization

To further simplify the image for subsequent segmentation, binarization was applied to convert the grayscale image into a binary format containing only black and white pixels, thereby reducing its complexity. A threshold value of 0.5 was selected for the binarization process. This process enhanced the contrast between the lung regions and the surrounding background, facilitating more efficient and accurate analysis in the following computational steps. After binarization, the lung area could be delineated more precisely. However, to further refine the lung boundaries and eliminate noise, morphological operations were employed to improve the structural integrity of the segmented regions. Morphological processing is a widely used technique in image analysis, particularly effective in enhancing specific image features. Among various operations, dilation and erosion are the most fundamental, with their definitions provided in Equations (2) and (3), respectively. The result of the image preprocessing step is presented in Figure 3d.

(2) A∘B=(A⊕B)⊖B

(3) A●B=(A⊖B)⊕B

### 2.2. Image Segmentation

**Figure 4:**
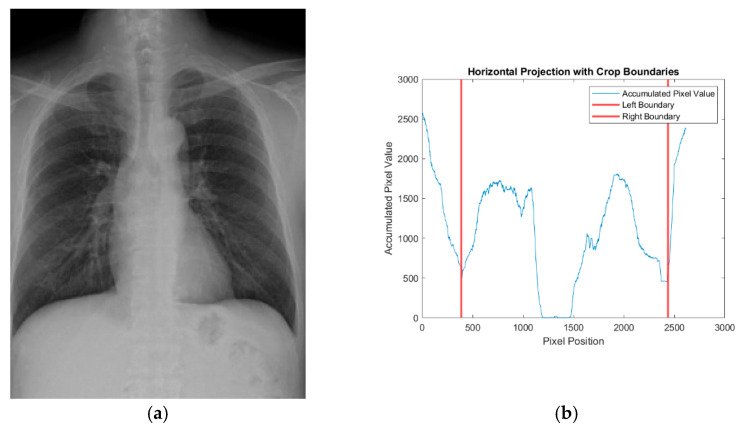

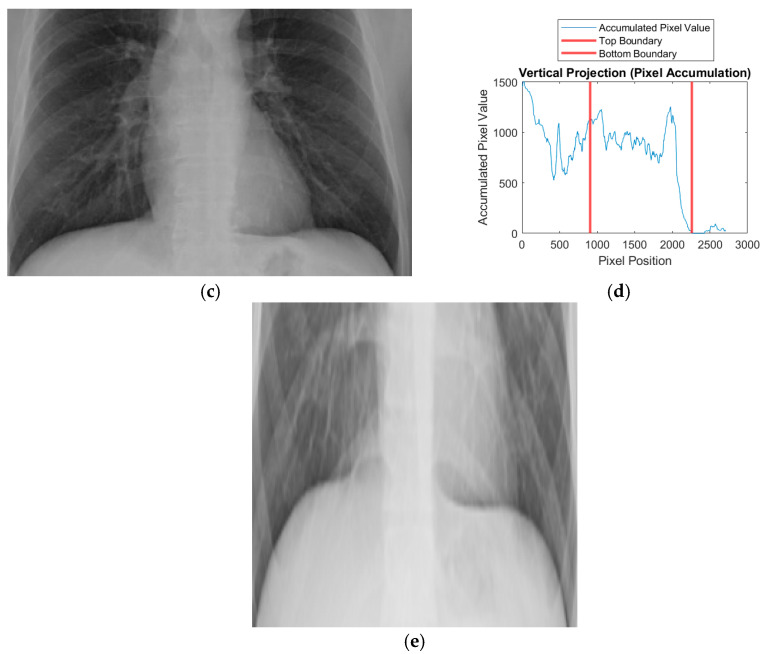
The results of the CXR image segmentation. (**a**) Vertical cropping image. (**b**) Vertical cumulative pixelmap. (**c**) Horizontal cropping image. (**d**) Horizontal cumulative pixelmap. (**e**) Final segmented and normalized image.

After binarization, the pixel values of the image were summed column by column to generate a vertical projection profile. This profile provides an intuitive view of pixel intensity distribution across the image and serves as the basis for determining suitable cropping boundaries. In this study, the vertical projection was used to identify the left and right cropping positions. Specifically, within the leftmost one-fifth of the image, the position with the lowest accumulated pixel value was selected as the candidate for the left boundary. Similarly, the rightmost one-fifth was analyzed to determine the right boundary based on the minimum pixel sum. Once both the left and right boundaries were identified, they were mapped back to the original CXR image to perform precise cropping, as shown in Figure 4a,b.

To further separate the lung region from surrounding structures, horizontal cropping was performed following the vertical cropping step. The horizontal cropping method followed a similar procedure to the vertical approach, in which pixel values were summed row by row to generate the cumulative vertical projection from top to bottom. To determine the upper boundary, the first row within the central one-third of the image whose total pixel value exceeded a predefined threshold was selected. This ensured accurate preservation of the upper margin of the lungs. The lower boundary was determined by identifying the row with the minimum accumulated pixel value in the lower half of the image, thereby preserving the complete structure of the costophrenic angle. Once the upper and lower boundaries were confirmed, they were mapped onto the CXR image that had already been cropped along the left and right boundaries, and vertical cropping was performed accordingly, as shown in Figure 4c,d. This method retains the main anatomical structures of the lungs while effectively excluding irrelevant peripheral regions, ensuring the integrity of the lung area and the removal of non-lung regions.

Additionally, to highlight the key features of pleural effusion, the lung region was further horizontally divided at the midpoint after completing both horizontal and vertical cropping, with only the lower half of the lung, from the heart to the costophrenic angle, being retained. The final cropped images were uniformly resized to 227×227 pixels to standardize the input dimensions for model training and ensure compatibility with the subsequent model requirements. The results of image segmentation and normalization are shown in Figure 4e.

### 2.3. Image Enhancement

In CXR images, pleural effusion typically occurred in the lower regions of the lungs and appeared as white or grayish-white areas, whereas healthy lung tissue was represented by relatively darker pixels. The visibility of pleural effusion on CXR images was influenced by various factors, including the extent, density, and location of the fluid. Therefore, accurately identifying pleural effusion was challenging. To address this issue, this study developed a feature enhancement algorithm for pleural effusion. The algorithm was applied to the cropped CXR images, representing the region of interest (ROI), and combined histogram stretching with Sobel gradient edge detection [21,22]. Histogram stretching improved image contrast, making the costophrenic angle more discernible, while Sobel edge detection emphasized the boundaries between tissues of different densities, thereby enhancing the clarity of pleural effusion features. In this way, both the visual characteristics and edge structures of pleural effusion in CXR images were effectively enhanced.

#### 2.3.1. Histogram Stretching

**Figure 5:**
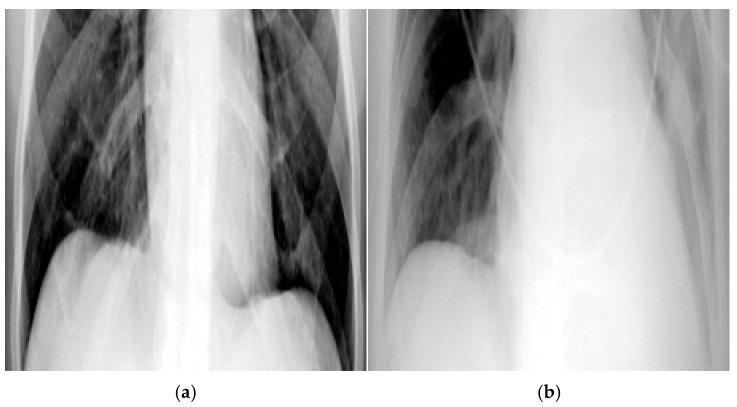
Results after applying histogram stretching. (**a**) Normal lung (**b**) Pleural effusion.

Enhanced imaging was necessary to better distinguish pleural effusion symptoms from surrounding lung contours. In CXR images, pleural effusion typically appeared as gray and blurred regions, while normal lung tissue remained relatively dark. This made accurate detection difficult, particularly in early stages. To improve image contrast, this study tested three enhancement methods: logarithmic transformation [23], Contrast Limited Adaptive Histogram Equalization (CLAHE) [24], and histogram stretching [25], with the aim of highlighting the costophrenic angle. Among these methods, histogram stretching demonstrated the most effective performance; therefore, this study employed histogram stretching as a computationally efficient enhancement technique, as shown in Figure 5. Histogram stretching effectively increased the brightness contrast between the symptomatic areas and adjacent normal tissues, making the effusion more visually distinguishable. This enhancement supported improved feature extraction by the CNN, thereby improving classification and detection accuracy.

Histogram stretching worked by identifying the minimum and maximum pixel intensity values in the input image and linearly mapping them to the full dynamic range. This transformation amplified subtle differences in pixel intensity, making previously indistinct features more pronounced. The detailed algorithmic steps are presented in Algorithm 1.
**Algorithm 1. Histogram Stretching.***Input* 
$I_{i}$
*: filtering input image.*
   $I_{o}$
*: Scaling constant.*
   $\left(K_{w},K_{h}\right)$
*: Dimensions of the input image.*
*Output* 
$I_{o}\;$
*: Transformed output image.*
    $I_{o}\left(x,y\right)=(I_{i}\left(x,y\right)-min)\times 255/(\max-min)$
*Hint: x* 
$∊\left[0\ldots K_{w}-1\right],\;n∊\left[0\ldots K_{h}-1\right]$
    *min, max: minimum and maximum pixel values in input image*


#### 2.3.2. Sobel Gradient Edge Detection

Since histogram stretching enhanced the visibility of the costophrenic angle but was insufficient to highlight the features of pleural effusion, this study further employed edge detection techniques to enhance pleural effusion characteristics. The performance of several methods, including Sobel [26,27], Sobel gradient, sharpening [28], and Canny edge detection [29], was evaluated in terms of their impact on subsequent model classification. Among these, the Sobel gradient demonstrated the best performance; therefore, it was selected in this study to further enhance the features of pleural effusion. Specifically, the Sobel gradient edge detection algorithm was adopted to identify edge contours within the CXR images. The Sobel operator computed the image intensity gradient at each pixel, indicating the direction of the greatest rate of change from light to dark, along with the magnitude of that change. It utilized two 3×3 convolution kernels, one designed to detect horizontal changes and the other for vertical changes. These kernels were convolved with the original image to approximate the gradient in each direction, which were then combined to compute the overall gradient magnitude. This method was particularly effective in medical imaging, as it emphasized the boundaries between different tissue regions while maintaining robustness against noise. The detailed computational steps of the Sobel gradient operator are provided in Algorithm 2.
**Algorithm 2. Sobel Gradient Edge Detection.***1.* *Apply Gaussian filter to reduce noise (optional pre-processing step).*$G\left(x,y\right)=\frac{1}{2\pi \sigma^{2}}exp\left(-\frac{\left(x^{2}+y^{2}\right)}{2\sigma^{2}}\right)$*2.* *Apply Sobel operators to compute horizontal and vertical gradients.*$Gx=\left[\left[-1,\;0,\;1\right],\;\left[-2,\;0,\;2\right],\;\left[-1,\;0,\;1\right]\right]\times I\left(x,y\right)$Gy=[[−1,−2,−1], [0, 0, 0], [1, 2, 1]]×I(x,y)*3.* *Calculate gradient magnitude.*$|G|=\sqrt{\left(Gx^{2}+Gy^{2}\right)}$*4.* *Calculate gradient direction (optional).*$\theta (x,y)={tan}^{-1}(Gy/Gx)$*5.* *Apply threshold to create binary edge map (optional).**If |G| > threshold, pixel is an edge**Otherwise, pixel is not an edge**6.* *Combine with original image (optional).**Result =*α×Original+β×Gradient_map

**Figure 6:**
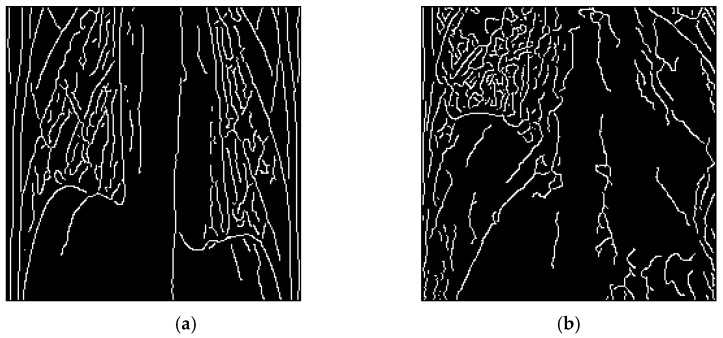
Results after applying image enhancement method. (**a**) Normal lung (**b**) Pleural effusion.

After applying the Sobel gradient edge detection, the resulting gradient map enhanced the visibility of the costophrenic angle. In normal lung tissue, the contour of the costophrenic angle appeared sharp and exhibited a steep gradient. However, in the presence of pleural effusion, this region became blurred. This distinct difference in gradient patterns enabled the model to better capture the characteristics of pleural effusion, thereby improving diagnostic accuracy. The results of image enhancement are shown in Figure 6. In the enhanced healthy lung images, the curvature from the pleura to the costophrenic angle follows a normal arc, as shown in Figure 6a. In contrast, for images containing pleural effusion, this expected curvature is absent due to fluid accumulation, as shown in Figure 6b.

### 2.4. CNN Training and Validation

With the rapid advancement of machine learning and artificial intelligence technologies, CNNs have been increasingly applied to image processing tasks and have demonstrated significant success in various image classification problems. In terms of model selection, this study evaluated several CNN architectures, including MobileNet-v3 [30], SqueezeNet [31], DarkNet19 [32], AlexNet [33], and EfficientNet-B0. Model performance was assessed using 5-fold cross-validation to ensure robustness and stability. The hardware and software platform used in this study is shown in Table 1. Among these, EfficientNet-B0 achieved the best performance and was therefore chosen as the backbone model. By leveraging clinically acquired CXR images, the proposed system was designed to be well-suited for practical implementation in real-world clinical settings. Among them, EfficientNet-B0 is a highly efficient CNN architecture that balances network depth, width, and input resolution to achieve optimal performance [34]. It employs a compound scaling method that simultaneously scales these dimensions, thereby improving accuracy while reducing computational cost. EfficientNet-B0 has shown strong performance in both general image classification and medical image analysis, effectively enhancing disease recognition accuracy and reducing the risk of overfitting.

During CNN training, hyperparameters played a crucial role in influencing the learning behavior and overall performance of the model. The learning rate, batch size, and epochs are among the most commonly adjusted hyperparameters. The learning rate controlled the step size for weight updates during training. Specifically, a higher learning rate accelerated model convergence but could miss the optimal solution, whereas a lower learning rate ensured more stable updates but required significantly more training time. In addition, the batch size defined how many samples were used to update the model parameters in each iteration of training. Larger batch sizes could accelerate training and provide smoother gradient estimates but required more memory. On the other hand, smaller batch sizes introduced more variance in updates, which could help avoid local minimum but might also lead to instability during training. Moreover, the number of epochs referred to how many times the model was trained on the entire training dataset. Although increasing the number of epochs allowed the model to learn more effectively from the data, it also increased the risk of overfitting. Therefore, selecting appropriate hyperparameters was essential to ensure effective model training. The specific hyperparameter settings used in this study are summarized in Table 2.

## 3. Results

The study procedures complied with ethical standards and were approved by the Institutional Review Board (IRB) of Chiayi Chang Gung Memorial Hospital (IRB No. 202301914B0). All CXR images used in this study were collected from adult patients (aged 18 and above), and the Chang Gung Medical Foundation Institutional Review Board approves the waiver of the participants’ consent. A total of 922 CXR images were included, comprising 461 images of normal lungs and 461 images diagnosed with pleural effusion. Among them, 80% (738 images) were used for model training, while the remaining 20% (184 images) were reserved for validation. The distribution of the training data is summarized in Table 3.

This section presents the experimental results of the proposed CNN approach for detecting pleural effusion in CXR images. The evaluation includes four main components: the performance metrics used in this study, a comparison of model performance with single-lung and whole-lung as inputs, an assessment of various image enhancement methods, and a comparative analysis of classification results across multiple CNN architectures. The following subsections describe these experiments in detail, highlighting the key findings and their implications for clinical application.

### 3.1. Performance Metrics

The primary objective was to reduce the workload of radiologists while minimizing the risk of misdiagnosis. Accuracy, precision, recall, and F1 score were adopted as evaluation metrics for the model’s performance, as they are commonly used in classification tasks. As shown in Table 4, the confusion matrix served as the basis for calculating the evaluation metrics defined in Equations (4)–(7).

(4) $$\mathrm{Accuracy}=\frac{\mathrm{TP}+\mathrm{TN}}{\mathrm{TP}+\mathrm{TN}+\mathrm{FP}+\mathrm{FN}}$$

(5) $$\mathrm{Precision}=\frac{\mathrm{TP}}{\mathrm{TP}+\mathrm{FP}}$$

(6) $$\mathrm{Recall}=\frac{\mathrm{TP}}{\mathrm{TP}+\mathrm{FN}}$$

(7) $$F1\;s\mathrm{core}=2\times \frac{\mathrm{Precision}\;\times \;\mathrm{Recall}}{\mathrm{Precision}+\mathrm{Recall}}$$

### 3.2. Comparison of Single-Lung and Whole-Lung Inputs

In this study, the performance of single-lung and whole-lung inputs was compared. The comparison results of single-lung and whole-lung inputs are shown in Table 5, where the single-lung input was further divided into left and right lung cases. The left lung achieved an accuracy of 66.30%, whereas the right lung achieved a higher accuracy of 73.37%. This indicated that the right lung outperformed the left lung, likely due to the influence of the heart’s position, which interfered with the model’s ability to detect left-sided pleural effusion.

Furthermore, the heart’s position made the imaging characteristics of the left lung more complex, making it more difficult to accurately capture symptomatic information. As a result, the average single-lung accuracy was 69.84%. In contrast, the whole-lung input achieved an accuracy of 84.24%, showing that pleural effusion features were more clearly represented in the whole-lung images. Based on these comparative results, the whole-lung image was ultimately selected as the CNN input for training and validation.

### 3.3. Evaluation of Different Image Enhancement Method

To effectively improve model accuracy, this study applied multiple image enhancement techniques to the cropped CXR images. The enhancement process was divided into two stages, targeting the costophrenic angle and pleural effusion features, respectively.

For the enhancement of costophrenic angle features, three methods were compared: logarithmic transformation, CLAHE, and histogram stretching, along with the unenhanced images. The training results for each method are summarized in Table 6. Histogram stretching achieved the best overall performance, with accuracy at 89.67%, precision at 90.06%, recall at 89.67%, and F1 score at 89.65%. The original images without enhancement achieved an accuracy of 87.50%. CLAHE yielded accuracy comparable to the original method, while logarithmic transformation performed the worst, with all evaluation metrics significantly lower than the other three approaches. Therefore, histogram stretching was selected as the enhancement method for costophrenic angle features in this stage.

Following the selection of histogram stretching for enhancing the costophrenic angle features, this study further evaluated multiple edge detection techniques to enhance the features of pleural effusion. The experiments were conducted on images that had been cropped and processed with histogram stretching. The evaluated methods included Sobel, Sobel gradient, sharpening, and Canny edge detection, and the results are presented in Table 7. As shown in Table 7, the Sobel gradient method achieved the best performance, with these four metrics all reaching 91.85%. Sharpening and Sobel yielded accuracies of 88.04% and 86.96%, respectively. In contrast, the Canny edge detection method had the lowest performance among the four methods, with all metrics at 83.15%. Based on these results, histogram stretching combined with the Sobel gradient method was ultimately selected as the image enhancement approach to improve the accuracy and performance for the model training.

### 3.4. The Classification Results of CNN

To further verify the stability of the proposed pleural effusion detection model, 5-fold cross-validation was conducted to evaluate multiple deep learning architectures. Five different CNN architectures were tested, including MobileNet-v3, SqueezeNet, DarkNet19, AlexNet, and EfficientNet-B0. As shown in Table 8, EfficientNet-B0 attained the best accuracy of 93.27%, closely followed by AlexNet at 92.74%. SqueezeNet and DarkNet19 recorded accuracy of 90.02% and 89.49%, respectively. In contrast, MobileNet-v3 yielded the lowest performance, with an accuracy of only 77.33%. Based on these findings, EfficientNet-B0 was selected as the final model for pleural effusion recognition in this study. Ultimately, the proposed system achieved an accuracy of 93.27% ± 0.02, a precision of 92.95% ± 0.03, a recall of 92.81% ± 0.02, and an F1 score of 92.79% ± 0.02. To further verify the clinical applicability of the model, the National Institutes of Health CXR Dataset [35] containing CXR images with pleural effusion was also used for testing. The results demonstrated an accuracy of 93.50 ± 0.03, a precision of 93.00 ± 0.02, and a recall of 93.00 ± 0.02, confirming the reliability of the system and its potential for clinical implementation.

To evaluate the contribution of the proposed system, a comparison was conducted with previous studies, as summarized in Table 9. The results presented a direct comparison between earlier pleural effusion detection methods and the approach developed in this study. The proposed method achieved the highest accuracy at 93.27%, representing an improvement of approximately 21.30% over Method [5], which achieved 77.00%. In terms of precision, Method [17] obtained the highest value at 99.00%, exceeding the proposed method by 6.05%. For recall, Method [16] and Method [14] achieved 100.00% and 94.70%, respectively, while the proposed method reached 92.81%, indicating potential for further improvement in this metric. However, in terms of the F1 score, which balances precision and recall, the proposed method outperformed all other approaches, demonstrating higher overall reliability. These notable improvements over prior methods indicated that the proposed system was capable of effectively capturing subtle features of pleural effusion in CXR images, thereby offering a more reliable diagnostic tool for clinical applications.

## 4. Discussion

Building on the comparative results presented in the previous section, this study specifically addressed the diagnostic challenges radiologists face in detecting pleural effusion, particularly in its early stages when the symptoms are subtle and often indistinguishable on standard CXR images. Analysis of our experimental results revealed that, while the detection accuracy for the right lung was comparable to that of whole-lung imaging, the accuracy for the left lung was adversely affected by its anatomical overlap with the heart. This observation motivated our focus on isolating the costophrenic angle to enhance detection accuracy.

To optimize feature extraction, a series of targeted image processing techniques were applied, including selective cropping and enhancement of the costophrenic angle. Among the evaluated enhancement methods, the combination of histogram stretching and Sobel gradient edge detection achieved the most significant improvement, with accuracy, precision, recall, and F1 score all exceeding 91.85%, outperforming other edge detection techniques. Regarding classification performance, EfficientNet-B0 demonstrated the highest overall effectiveness, achieving an accuracy of 93.27%, which was substantially higher than other deep learning models tested. Compared with previous studies, the proposed method achieved a 93.27% accuracy, representing an improvement of 21.30%, and obtained the highest model reliability as reflected in the top-performing F1 score.

These improvements can be attributed to the integrated approach of costophrenic angle cropping, advanced image enhancement, and edge detection, which collectively amplified pleural effusion features, thereby facilitating more efficient and accurate model training. In contrast, methods [5,15,16,17] relied solely on CNN-based classification without incorporating image preprocessing or cropping methods. However, it should be noted that the dataset used in our study for training and validation was collected from a Chiayi Chang-Gung Memorial Hospital during the study period, which differs from the datasets employed in methods [5,15,16,17]. This discrepancy may limit the fairness of direct performance comparisons. Future research will focus on further improving the model’s accuracy and robustness. Compared with previous studies, the proposed system still has room for improvement in recall performance, which may be influenced by hyperparameter settings during training or the limited size of the dataset. To address this, future work will combine multiple publicly available datasets for model training and validation. This approach is expected not only to expand the dataset for training but also to enhance the model’s applicability in clinical practice. In addition, future research plans to incorporate Generative Adversarial Networks (GANs) for image augmentation, comparing their performance and computational cost with traditional preprocessing methods. This approach is expected to reduce decision-making time while providing more comprehensive support for medical diagnosis and contributing to improved pulmonary healthcare services. Overall, the findings of this study demonstrate that a carefully designed combination of anatomical region selection, targeted image enhancement, and deep learning classification can substantially improve the detection of pleural effusion in CXR images, offering a clinically valuable and computationally efficient tool for early diagnosis and treatment planning. In the future, this system could be integrated with existing Picture Archiving and Communication Systems (PACS) to provide physicians and patients with preliminary identification results of pleural effusion, thereby enhancing clinical decision-making and fostering better communication between physicians and patients.

## 5. Conclusions

This study proposed an automated pleural effusion detection system that achieved significant advancements in diagnosis through optimized image preprocessing and deep learning models. The system first performed precise cropping of CXR images to preserve the critical costophrenic angles, followed by image enhancement using histogram stretching and Sobel gradient edge detection. The proposed preprocessing workflow proved more effective than other approaches, increasing the accuracy from 87.50% without enhancement to 91.85%, thereby confirming that image enhancement can effectively improve the visibility of key features and provide a solid foundation for the subsequent classification stage.

In the classification step, 5-fold cross-validation was employed to evaluate the performance of multiple CNN architectures. EfficientNet-B0 was ultimately selected as the classification model, achieving an accuracy of 93.27%. Compared with previous methods, the proposed system improved accuracy by approximately 21.30%, with both precision and recall exceeding 92.00%, demonstrating reliable detection capabilities for both positive and negative pleural effusion cases.

Overall, by integrating precise cropping with efficient image enhancement, the proposed system substantially improved the accuracy and robustness of pleural effusion detection, showing strong potential for clinical application. Future study will focus on expanding the dataset and further enhancing image preprocessing techniques to continually improve the model’s practicality and reliability in real-world clinical environments.

## Figures and Tables

**Table 1:** The hardware and software platform used in this study.

Hardware Platform	Version
CPU	Intel^®^ Core™ i5-12400
GPU	NVIDIA GeForce RTX™ 3060 Ti
Software platform	Version
Matlab	2023a

**Table 2:** The hyperparameters used in CNN model training.

Hyperparameter	Value
Learning Rate	0.00001
Batch Size	32
Epochs	20

**Table 3:** The distribution of the training and validation database.

Database	Training	Validation	Total
Normal Lung	369	92	461
Pleural Effusion	369	92	461

**Table 4:** An example of the confusion matrix.

		Ground Truth Value	
		True	False
Predicted Value	True	$T_{p}$ (True Positive)	$F_{p}$ (False Positive)
	False	$F_{n}$ (False Negative)	$T_{n}$ (True Negative)

**Table 5:** Performance comparison of using CNN to train single-lung and whole-lung pleural effusion classification.

	Left Lung	Right Lung	Average Lungs	Whole Lungs
Accuracy	66.30%	73.37%	69.84%	84.24%
Precision	79.87%	82.62%	81.25%	86.97%
Recall	66.30%	73.37%	69.84%	84.24%
F1 Score	61.99%	71.34%	66.67%	83.94%

**Table 6:** Comparison between the case without image enhancement and various image enhancement methods.

	Original	Log Transform	CLAHE	Histogram Stretch
Accuracy	87.50%	64.13%	87.50%	89.67%
Precision	88.52%	77.38%	88.04%	90.06%
Recall	87.50%	64.13%	87.50%	89.67%
F1 Score	87.42%	59.19%	87.46%	89.65%

**Table 7:** The comparison between various edge detection.

	Histogram Stretch	Histogram Stretch + Canny	Histogram Stretch + Sharpen	Histogram Stretch + Sobel Gradient
Accuracy	89.67%	83.15%	88.04%	91.85%
Precision	90.06%	83.16%	88.50%	91.86%
Recall	89.67%	83.15%	88.04%	91.85%
F1 Score	89.65%	83.15%	88.01%	91.85%

**Table 8:** The result with 5-fold cross-validation results of different CNN architectures.

	Mobilenet_v3	Squeezenet	Darknet19	Alexnet	Efficientnet_b0
Accuracy	77.53%	90.02%	89.49%	92.74%	93.27%

**Table 9:** Comparison of Deep Learning Methods for Pleural Effusion Detection.

	Method in [17]	Method in [5]	Method in [16]	Method in [14]	Method in [15]	This Work
Accuracy (%)	83.00	77.00	91.00	87.70	87.18	93.27
Precision (%)	99.00	82.25	84.21	85.30	N/A	92.95
Recall (%)	75.00	91.50	100.00	94.70	N/A	92.81
F1 score (%)	75.00	82.00	91.43	86.00	N/A	92.79

## Data Availability

The datasets presented in this article are not readily available because they are part of an ongoing study and will be made available only after the completion of data collection and analysis. Requests to access the datasets should be directed to the corresponding authors at simon-chi@mail.mcut.edu.tw or tsungychen@fcu.edu.tw.
